# Routine metabolic and nutrition-related composite indices for identifying adverse cardiovascular-kidney-metabolic phenotypes: a cross-sectional study

**DOI:** 10.3389/fnut.2026.1872579

**Published:** 2026-08-21

**Authors:** Jing Lin, Guangre Xu, FuLi Chen, Hao Sun, Zhiqin Yu, Yuantao Qi

**Affiliations:** 1 Department of Cardiology, Sichuan Provincial People's Hospital, University of Electronic Science and Technology of China, Chengdu, China; 2 Department of Gastroenterology, Sichuan Provincial People's Hospital, University of Electronic Science and Technology of China, Chengdu, China; 3 Qingdao Municipal Hospital, University of Health and Rehabilitation Sciences, Qingdao, China; 4 Shandong Cancer Hospital and Institute, Shandong First Medical University and Shandong Academy of Medical Sciences, Jinan, China

**Keywords:** atherogenic index of plasma, cardiovascular-kidney-metabolic syndrome, machine learning, NHHR, systemic immune-inflammation index, TyG index, UHR

## Abstract

**Background:**

Cardiovascular-kidney-metabolic (CKM) syndrome provides an integrated framework for capturing the overlap of adiposity, metabolic dysfunction, kidney impairment, and cardiovascular risk. Whether low-cost composite biomarkers derived entirely from routine health examinations and brief questionnaires can identify adverse CKM phenotypes in preventive-care populations remains insufficiently characterized.

**Methods:**

We conducted a single-center, hospital-based cross-sectional study of adults who underwent routine health examinations at the Health Examination Center of the Second Affiliated Hospital of Shandong First Medical University between 2024 and 2025. Participants were included in the complete-case analysis based on complete data availability. Demographic, lifestyle, anthropometric, blood pressure, complete blood count, and fasting biochemical variables were used to construct anthropometric, insulin-resistance, lipid-metabolic, inflammatory, and nutrition-related indices. The primary outcome was prevalent adverse CKM phenotype, defined as CKM stage > = 2. Multivariable logistic regression, receiver operating characteristic analysis, restricted cubic spline modeling, and internally validated machine-learning models were used to evaluate associations, discrimination, non-linearity, and model interpretability.

**Results:**

Among 1,226 participants (mean [SD] age, 47.42 [13.14] years; 631 men [51.47%]), 718 (58.56%) had adverse CKM phenotype. CKM stage 2 accounted for most adverse CKM cases (693/718, 96.52%), whereas stages 3 and 4 were uncommon (9 [0.73%] and 16 [1.31%] of the total sample, respectively). After adjustment for age, sex, smoking status, alcohol status, exercise frequency, and sleep duration, the strongest associations were observed for the atherogenic index of plasma (AIP; odds ratio [OR] per 1-SD increase, 6.70; 95% CI, 5.24–8.56; *p* < 0.01), triglyceride-glucose (TyG) index (OR, 6.38; 95% CI, 5.00–8.13; *p* < 0.01), non-HDL-C to HDL-C ratio (OR, 4.97; 95% CI, 3.86–6.41; *p* < 0.01), TyG-BMI (OR, 4.53; 95% CI, 3.67–5.58; *p* < 0.01), and TyG-WHtR (OR, 4.08; 95% CI, 3.33–5.01; *p* < 0.01). AIP and TyG showed similarly high single-index discrimination (AUC, 0.87 for both; *p* < 0.01). In the held-out testing set, the random forest model achieved the best internal performance (AUC, 0.89; 95% CI, 0.85–0.92; *p* < 0.01; PR-AUC, 0.94; accuracy, 0.82).

**Conclusion:**

In this single-center cross-sectional routine health examination population, adverse CKM phenotype was common but predominantly represented stage 2 disease. AIP and TyG were similarly high-performing low-cost summary markers of prevalent CKM-related metabolic risk. These findings suggest that routine composite indices may help summarize and preliminarily stratify prevalent CKM risk in health examination settings, pending external and longitudinal validation; they should not be interpreted as evidence of readiness for clinical deployment or prediction of future events.

## 1 Introduction

Cardiovascular-kidney-metabolic (CKM) syndrome has recently been formalized by the American Heart Association as a multiorgan construct that captures the reciprocal biological and clinical interactions among adiposity, dysglycemia, atherogenic dyslipidemia, chronic kidney disease, and cardiovascular disease (1–3). The clinical value of this framework lies in its staging logic: early metabolic and renal abnormalities can be recognized before irreversible cardiovascular complications develop. For hospital-based health examination programs, CKM staging offers a clinically intuitive structure for transforming routine screening data into integrated cardiometabolic risk information.

The burden of CKM-related disorders is particularly relevant in hospital-based preventive-care populations, where participants frequently have subclinical combinations of elevated blood pressure, central obesity, dyslipidemia, hyperuricemia as a related comorbidity, and impaired glycemic control. Traditional screening and electronic health record workflows can directly identify stage-defining abnormalities when all required components are available. Therefore, the clinical gap addressed by this study is not to replace formal CKM staging, but to determine whether low-cost composite indices can summarize prevalent CKM-related metabolic burden and help prioritize individuals for more complete clinical review in health examination settings.

Several routine-data-derived indices have gained attention as surrogates for insulin resistance, visceral adiposity, lipid remnant burden, and low-grade inflammation. The triglyceride-glucose (TyG) index was proposed as a simple marker of insulin resistance using fasting triglycerides and glucose (4, 5). Extensions such as TyG-BMI and TyG-WHtR combine glycemic-lipid stress with general or central adiposity (6, 7). The body roundness index (BRI) and waist-to-height ratio (WHtR) further represent geometry-based measures of central adiposity that can be calculated from routine anthropometric data (8–10). These indices are attractive for health examination settings because they require no insulin assay, imaging quantification, or specialized inflammatory testing.

Beyond insulin resistance and adiposity, lipid-derived, inflammation-derived, and nutrition-immune markers may capture complementary CKM dimensions. The atherogenic index of plasma (AIP), calculated as log10 (triglycerides/HDL-C), reflects atherogenic dyslipidemia and lipoprotein particle characteristics (11, 12). The non-HDL-C/HDL-C ratio (NHHR) integrates cholesterol contained in atherogenic particles relative to antiatherogenic HDL-C (13, 14). Uric acid-to-HDL-C ratio (UHR) links purine metabolism, oxidative stress, and dyslipidemia (15, 16). Complete blood count-derived indices, including systemic immune-inflammation index (SII), systemic inflammation response index (SIRI), and hemoglobin-albumin-lymphocyte-platelet (HALP) score, provide low-cost proxies of inflammatory and nutrition-immune status (17–21). Although HALP is less directly linked to CKM staging than lipid-insulin indices, it may reflect nutritional reserve, systemic inflammation, and immune-metabolic stress that accompany adverse cardiometabolic phenotypes.

Machine-learning methods can accommodate non-linear relationships and correlated predictors, but their clinical usefulness depends on transparent reporting, rigorous internal validation, adequate sample-size considerations, and interpretable output (22–27). Current TRIPOD+AI guidance emphasizes clarity in model development and performance evaluation when regression or machine-learning models are used in health prediction research (22). For cross-sectional health examination data, explainable models may identify the most informative low-cost signals while avoiding the overinterpretation of black-box predictions.

We therefore established a single-center hospital-based cross-sectional cohort consisting of individuals who underwent routine health examinations at the Health Examination Center of the Second Affiliated Hospital of Shandong First Medical University between 2024 and 2025. Using questionnaire data, anthropometric measurements, blood pressure, complete blood counts, and routine biochemical tests, we evaluated the association and discrimination of multiple composite indices for prevalent adverse CKM phenotype. This study focused on identifying prevalent CKM stage > = 2 phenotype, not predicting future CKM progression or cardiovascular-kidney outcomes.

## 2 Materials and methods

### 2.1 Study design and participants

**Figure 1:**
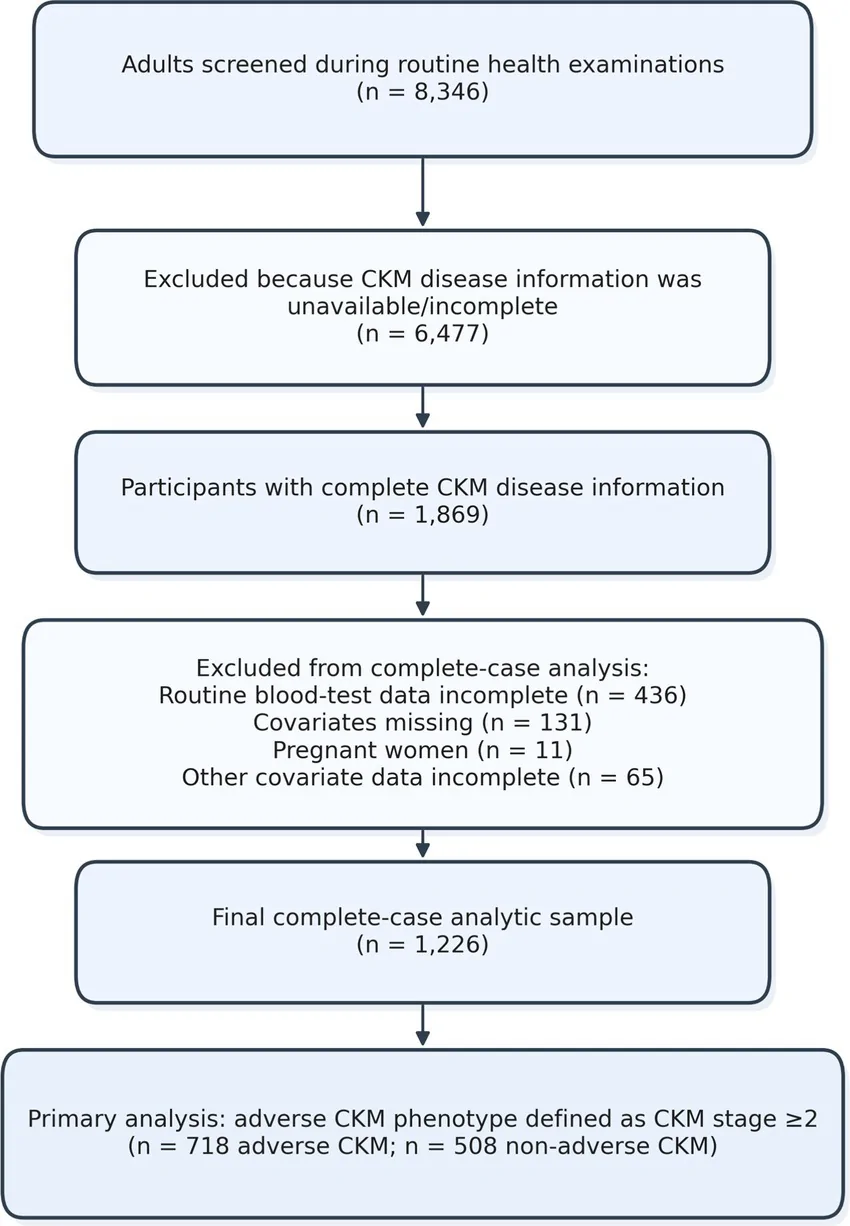
Participant flow diagram.

This single-center, hospital-based cross-sectional study analyzed individuals who underwent routine health examinations at the Health Examination Center of the Second Affiliated Hospital of Shandong First Medical University between 2024 and 2025. Participants were not randomly sampled; eligible records were included based on complete data availability for CKM disease information, routine blood testing, and prespecified covariates. A total of 8,346 examinees were screened. Of these, 1,869 had complete CKM disease information, and 6,477 were excluded because CKM disease information was unavailable or incomplete. We further excluded participants with incomplete routine blood-test data (*n* = 436), missing covariates (*n* = 131), pregnancy (*n* = 11), and other incomplete covariate data (*n* = 65), resulting in a final complete-case analytic sample of 1,226 participants. The participant flow is shown in Figure 1. The analytic dataset contained one record per participant. Questionnaire information was combined with routine anthropometric measurements, blood pressure, complete blood count, and fasting biochemical testing (28).

### 2.2 Data sources and measurements

Demographic and questionnaire variables included age, sex, smoking status, alcohol status, exercise frequency, and sleep duration. Anthropometric and clinical measurements included height, weight, waist circumference, systolic blood pressure, and diastolic blood pressure. Laboratory variables included fasting plasma glucose, HbA1c, total cholesterol, triglycerides, HDL-C, LDL-C, serum creatinine, eGFR, uric acid, neutrophil count, lymphocyte count, monocyte count, platelet count, hemoglobin, and albumin. CKM-related diagnoses were assigned by physicians during routine health examinations according to the operational definitions summarized in Supplementary Table 1, and the research team extracted these physician diagnoses and corresponding variables from the examination records. Albuminuria or urinary albumin-to-creatinine ratio (UACR) was not available in the extracted dataset. Hyperuricemia was recorded as an additional cardiometabolic comorbidity and was defined as serum uric acid >420 μmol/L regardless of sex; it was not used as an independent AHA CKM stage-defining criterion.

### 2.3 Variable construction

Composite indices were reconstructed from raw variables to ensure reproducibility, and all formulae were prespecified according to published definitions. BMI was calculated as weight (kg) divided by height squared (m^2^) (29). WHtR was calculated as waist circumference divided by height, using identical length units (9, 10). The TyG index was calculated as ln [fasting triglycerides (mg/dL) × fasting plasma glucose (mg/dL)/2] (4, 5). TyG-BMI and TyG-WHtR were calculated by multiplying TyG by BMI and WHtR, respectively (6, 7).

BRI was calculated as 364.2–365.5 × √ {1 − [(waist circumference/2π)^2^/(0.5 × height)^2^]}, with waist circumference and height expressed in consistent units.8 AIP was calculated as log10(triglycerides/HDL-C) (11, 12). NHHR was calculated as (total cholesterol − HDL-C)/HDL-C.13,14 UHR was calculated as serum uric acid divided by HDL-C (15, 16).

Inflammation- and nutrition-related indices were derived from the complete blood count and albumin values. SII was calculated as platelet count × neutrophil count/lymphocyte count (17, 18). SIRI was calculated as neutrophil count × monocyte count / lymphocyte count (18, 19). HALP was calculated as hemoglobin × albumin × lymphocyte count/platelet count (20, 21). Thus, all candidate indices were obtainable from routine anthropometry, fasting biochemistry, and complete blood count data without specialized assays. A concise list of formulas, required variables, units, and intended biological interpretation is provided in Supplementary Table 2.

### 2.4 Outcome definition

The primary outcome was prevalent adverse CKM phenotype, defined as CKM stage > = 2. CKM staging followed the American Heart Association conceptual framework (1, 2), but it was operationalized using available health examination records and physician-diagnosed conditions rather than independently reclassifying every AHA component from raw data. The extracted dataset did not include albuminuria/UACR or an independent imaging-defined fatty liver variable; therefore, the AHA framework could not be applied with every possible component. Stage 0 indicated no CKM risk factors; stage 1 indicated excess/dysfunctional adiposity or prediabetes without other major CKM risk factors; stage 2 indicated metabolic risk factors or CKD, including hypertension, hypertriglyceridemia/dyslipidemia, metabolic syndrome, diabetes, or moderate-to-high-risk CKD; stage 3 indicated CKM with subclinical CVD or very-high-risk CKD; and stage 4 indicated CKM with clinical CVD. Full operational criteria and available-data considerations are summarized in Supplementary Table 1. Hyperuricemia was not treated as an independent criterion for stage 2 or higher and was retained only as a descriptive comorbidity/related cardiometabolic component.

### 2.5 Statistical analysis

Continuous variables are presented as mean (SD), and categorical variables as number (%), as prespecified. Between-group comparisons used Welch *t*-tests for continuous variables and chi-square or Fisher exact tests for categorical variables. All displayed inferential results include *p*-values, with *p* < 0.05 considered statistically significant. Effect estimates and descriptive values were rounded to two decimal places. Missing data were handled by complete-case analysis after the exclusions shown in Figure 1; no imputation was performed.

**Figure 2:**
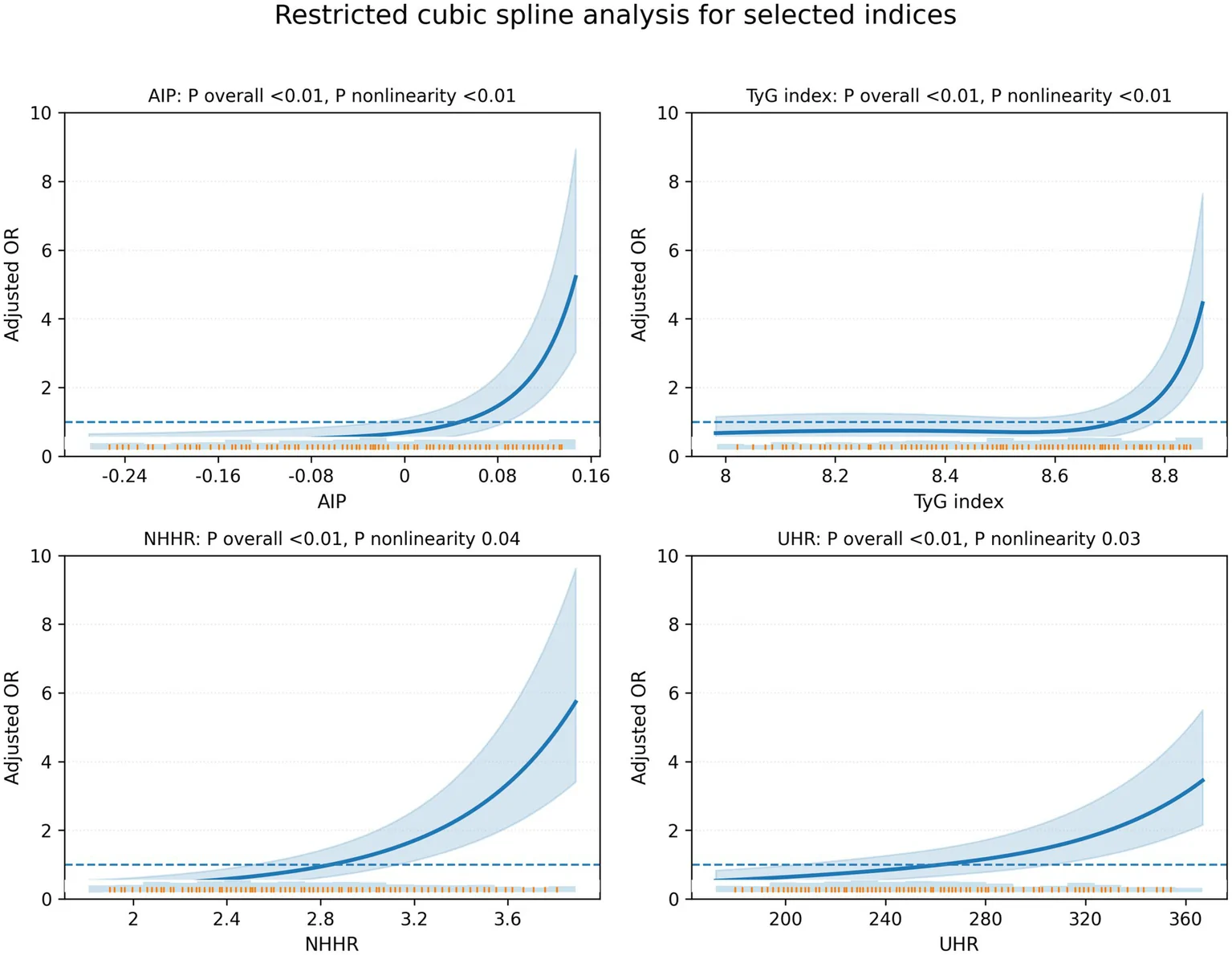
Restricted cubic spline curves for selected indices. Curves are centered at the median and displayed with 95% CIs over the data-dense central range of each index to keep the curves and confidence bands within a reasonable visual scale. Embedded histograms and rug plots show where observations were concentrated. Overall and non-linearity *p*-values are based on the full covariate-adjusted spline models reported in Table 1; the curves should not be interpreted as defining discrete clinical thresholds.

Associations between each index and adverse CKM phenotype were assessed using logistic regression. Indices were standardized, and odds ratios (ORs) were reported per 1-SD increment. Model 1 adjusted for age and sex. Model 2 further adjusted for smoking status, alcohol status, exercise frequency, and sleep duration. Each index was modeled separately under the same covariate-adjustment strategy to avoid unstable interpretation from mathematically related and highly intercorrelated indices. Logistic-regression diagnostics included inspection of continuous-predictor linearity using restricted cubic splines, assessment of influential extreme values by distributional review and 1st/99th percentile winsorization sensitivity analyses, and exploratory variance inflation factor (VIF) diagnostics when all indices were entered together. For Figure 2, the RCS curves were centered at the median and displayed with 95% CIs over the data-dense central range of each index. This visualization was used to keep the curves and confidence bands within a reasonable scale and to avoid overemphasis of sparse tail observations; the formal overall and non-linearity *p*-values were still based on the full covariate-adjusted spline models and are reported in Table 1. Discrimination of individual indices was assessed using ROC curves and the area under the curve (AUC). AUC comparisons among selected top-performing indices were performed using DeLong tests. Optimal cutoffs were derived using the Youden index but were considered internally derived and exploratory rather than clinically actionable thresholds. Because multiple correlated indices and exploratory analyses were assessed, results were interpreted as hypothesis-generating; no formal multiplicity correction was applied. Sex-stratified ROC analyses were performed as sensitivity analyses because sex distribution differed between CKM groups. Model development and validation were interpreted according to established prediction-model reporting, validation, and sample-size principles (22–25).

**Table 1:** Restricted cubic spline *p*-values.

Index	P overall	*P* non-linearity
AIP	<0.01	<0.01
TyG index	<0.01	<0.01
NHHR	<0.01	0.03
UHR	<0.01	0.03

Models were adjusted for age, sex, smoking status, alcohol status, exercise frequency, and sleep duration.

### 2.6 Machine-learning analysis

The machine-learning feature set included age, sex, smoking status, alcohol status, exercise frequency, sleep duration, and the 12 composite indices. Direct outcome labels and CKM component diagnoses were excluded from prediction features to reduce circularity. Participants were split into training and testing sets in a stratified 70:30 ratio with a fixed random seed. Categorical variables were one-hot encoded, and continuous variables were standardized for LASSO logistic regression. Candidate models included LASSO logistic regression, random forest, gradient boosting, XGBoost when available, and LightGBM when available. Hyperparameters were prespecified parsimoniously rather than extensively optimized: LASSO used L1-penalized logistic regression with C = 1.0 and the liblinear solver; random forest used 500 trees with square-root feature selection; gradient boosting used 100 estimators, learning rate 0.1, and maximum depth 3; XGBoost and LightGBM, when available, used 300 estimators, learning rate 0.05, subsampling 0.8, and column subsampling 0.8. No external validation, repeated cross-validation, or *post hoc* hyperparameter optimization was performed; therefore, model results were interpreted as exploratory internal-validation findings. Model selection was based on testing-set AUC, and performance was summarized using AUC, PR-AUC, accuracy, sensitivity, specificity, precision, F1-score, and Brier score. AUC confidence intervals and *p*-values were calculated using large-sample variance approximations. Model interpretability was evaluated using model-derived feature importance for the best-performing algorithm. Machine-learning analyses were reported with reference to biomedical machine-learning guidance and clinical AI implementation principles (22, 26, 27).

## 3 Results

### 3.1 Participant characteristics

**Figure 3:**
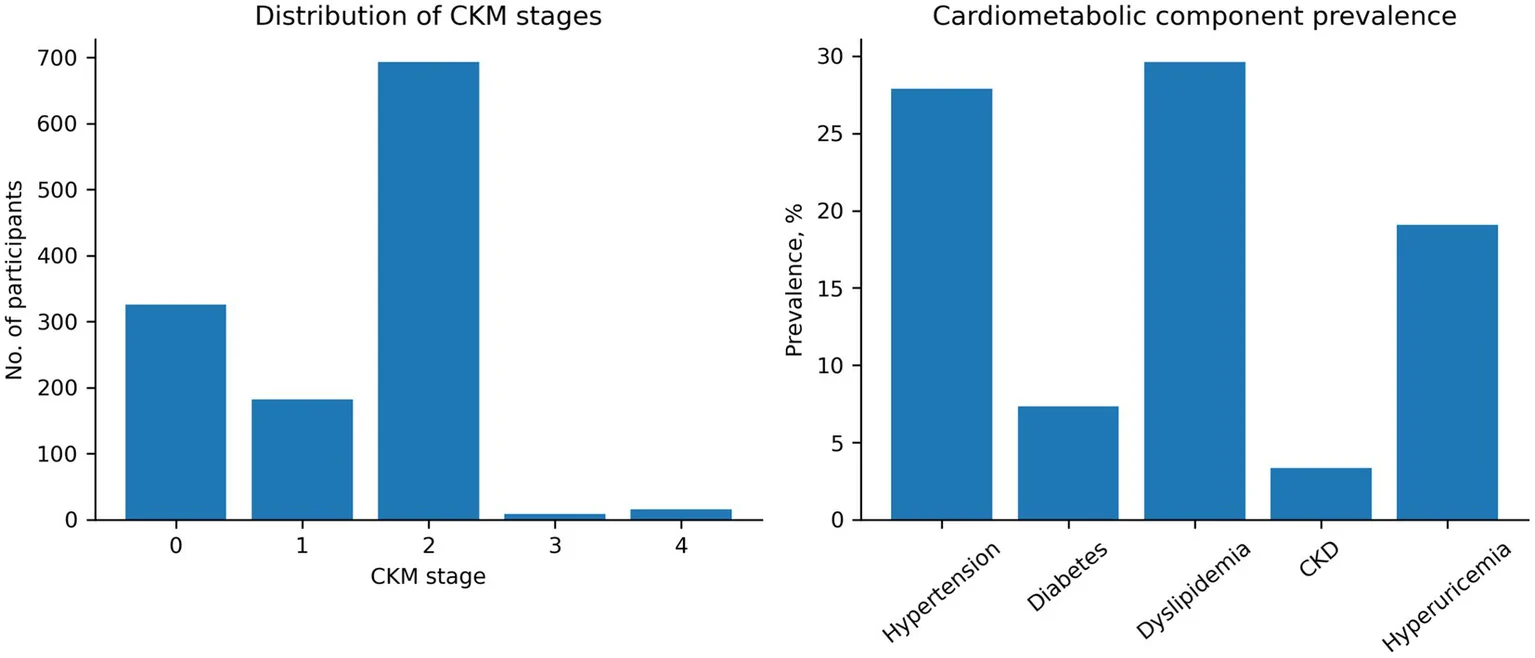
Distribution of CKM stages and prevalence of major cardiometabolic components.

A total of 1,226 participants were included in the complete-case analysis after screening and exclusions (Figure 1). The mean age was 47.42 (13.14) years, and 631 participants (51.47%) were men. The CKM stage distribution was as follows: stage 0, 326 participants (26.59%); stage 1, 182 (14.85%); stage 2, 693 (56.53%); stage 3, 9 (0.73%); and stage 4, 16 (1.31%). Overall, 718 participants (58.56%) had adverse CKM phenotype, whereas 508 (41.44%) were classified as non-adverse CKM. Importantly, adverse CKM was predominantly stage 2 (693/718, 96.52%), whereas stages 3 and 4 were rare in this health examination cohort. The prevalences of major cardiometabolic components were hypertension, 342 (27.90%); diabetes, 90 (7.34%); dyslipidemia, 363 (29.61%); CKD, 41 (3.34%); and hyperuricemia, 234 (19.09%). The CKM stage distribution and component prevalence are presented in Figure 3.

Compared with the non-adverse CKM group, participants with adverse CKM were older and had higher weight, waist circumference, systolic and diastolic blood pressure, fasting plasma glucose, HbA1c, triglycerides, LDL-C, creatinine, uric acid, BMI, WHtR, TyG, TyG-BMI, TyG-WHtR, BRI, AIP, NHHR, UHR, SII, and SIRI (all relevant *p* < 0.01). HDL-C, eGFR, albumin, and HALP were lower in the adverse CKM group. Sleep duration did not differ materially between groups (*p* = 0.68). Detailed baseline characteristics are shown in Table 2.

**Table 2:** Baseline characteristics according to adverse CKM phenotype.

Characteristic	Overall	Non-adverse CKM	Adverse CKM	*P*-value
Overall	1,226	508	718	
Age, years	47.42 (13.14)	41.99 (11.42)	51.26 (12.93)	<0.01
Sleep duration, h/day	6.97 (0.93)	6.98 (0.93)	6.96 (0.94)	0.68
Height, cm	164.79 (8.13)	163.71 (8.00)	165.56 (8.14)	<0.01
Weight, kg	65.92 (9.93)	61.48 (8.41)	69.06 (9.72)	<0.01
Waist circumference, cm	84.13 (8.18)	79.95 (6.76)	87.08 (7.81)	<0.01
Systolic blood pressure, mmHg	123.93 (15.26)	116.04 (11.85)	129.51 (14.94)	<0.01
Diastolic blood pressure, mmHg	76.86 (9.18)	72.84 (7.77)	79.71 (9.05)	<0.01
Fasting plasma glucose, mmol/L	5.34 (0.67)	4.99 (0.52)	5.59 (0.65)	<0.01
HbA1c, %	5.40 (0.42)	5.19 (0.34)	5.55 (0.40)	<0.01
Total cholesterol, mmol/L	5.07 (0.83)	4.68 (0.65)	5.35 (0.83)	<0.01
Triglycerides, mmol/L	1.68 (0.93)	1.09 (0.32)	2.09 (0.99)	<0.01
HDL-C, mmol/L	1.30 (0.24)	1.41 (0.20)	1.22 (0.23)	<0.01
LDL-C, mmol/L	2.84 (0.63)	2.60 (0.57)	3.01 (0.61)	<0.01
Creatinine, μmol/L	68.27 (12.97)	64.28 (11.44)	71.09 (13.25)	<0.01
eGFR, mL/min/1.73 m^2^	105.48 (14.82)	110.92 (12.57)	101.63 (15.09)	<0.01
Uric acid, μmol/L	342.04 (67.30)	321.70 (62.36)	356.44 (67.00)	<0.01
Neutrophils, 10^9^/L	3.53 (0.77)	3.36 (0.72)	3.65 (0.78)	<0.01
Lymphocytes, 10^9^/L	1.85 (0.45)	1.86 (0.45)	1.85 (0.45)	0.7
Monocytes, 10^9^/L	0.40 (0.13)	0.38 (0.12)	0.42 (0.14)	<0.01
Platelets, 10^9^/L	242.01 (49.67)	237.00 (49.48)	245.56 (49.52)	<0.01
Hemoglobin, g/L	141.74 (14.48)	139.99 (13.75)	142.97 (14.85)	<0.01
Albumin, g/L	44.94 (2.45)	45.26 (2.55)	44.72 (2.35)	<0.01
BMI, kg/m^2^	24.19 (2.53)	22.88 (2.12)	25.12 (2.39)	<0.01
WHtR	0.51 (0.05)	0.49 (0.05)	0.53 (0.05)	<0.01
TyG index	8.74 (0.58)	8.32 (0.37)	9.03 (0.52)	<0.01
TyG-BMI	212.25 (32.86)	190.74 (22.18)	227.47 (30.61)	<0.01
TyG-WHtR	4.48 (0.66)	4.08 (0.47)	4.77 (0.62)	<0.01
BRI	3.62 (1.00)	3.20 (0.84)	3.93 (1.00)	<0.01
AIP	0.06 (0.27)	−0.13 (0.17)	0.20 (0.24)	<0.01
NHHR	3.07 (1.18)	2.38 (0.64)	3.55 (1.24)	<0.01
UHR	276.57 (91.68)	235.05 (64.04)	305.95 (96.83)	<0.01
SII	477.11 (156.17)	442.22 (137.74)	501.79 (163.65)	<0.01
SIRI	0.80 (0.36)	0.71 (0.30)	0.87 (0.38)	<0.01
HALP	51.11 (18.58)	52.50 (19.91)	50.12 (17.53)	0.03
Sex				<0.01
Female	595 (48.53)	302 (59.45)	293 (40.81)	
Male	631 (51.47)	206 (40.55)	425 (59.19)	
Smoking status				<0.01
Never	876 (71.45)	394 (77.56)	482 (67.13)	
Former	75 (6.12)	22 (4.33)	53 (7.38)	
Current	275 (22.43)	92 (18.11)	183 (25.49)	
Alcohol status				<0.01
Never	663 (54.08)	300 (59.06)	363 (50.56)	
Occasional	347 (28.30)	140 (27.56)	207 (28.83)	
Regular	168 (13.70)	51 (10.04)	117 (16.30)	
Heavy	48 (3.92)	17 (3.35)	31 (4.32)	
Exercise frequency				0.21
<1/week	381 (31.08)	144 (28.35)	237 (33.01)	
1–2/week	362 (29.53)	160 (31.50)	202 (28.13)	
3–4/week	321 (26.18)	141 (27.76)	180 (25.07)	
≥5/week	162 (13.21)	63 (12.40)	99 (13.79)	

Data are presented as mean (SD) or No. (%). *P*-values were calculated using Welch *t*-tests for continuous variables and chi-square or Fisher exact tests for categorical variables. CKM indicates cardiovascular-kidney-metabolic; HDL-C, high-density lipoprotein cholesterol; eGFR, estimated glomerular filtration rate.

### 3.2 Variable construction and candidate biomarkers

**Figure 4:**
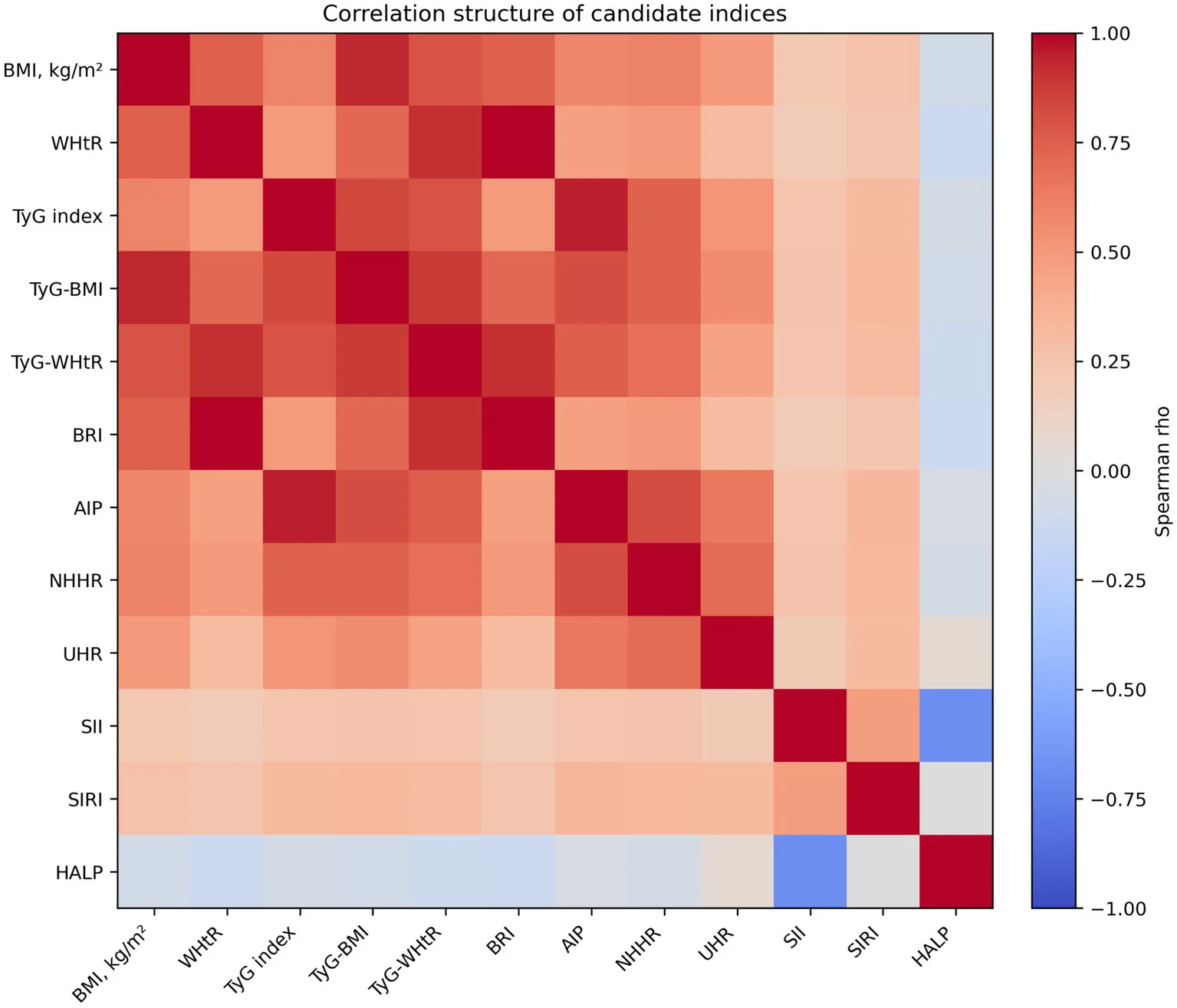
Spearman correlation heatmap of candidate composite indices.

All candidate indices were derived from routinely collected variables. Formula reconstruction showed negligible discrepancies from the uploaded calculated fields, consistent with rounding during data entry. The formulae and required variables for each candidate index are described in the Variable Construction subsection of the Methods and in Supplementary Table 2. The pairwise Spearman correlation structure of the candidate composite indices is shown in Figure 4. High correlations were expected because several indices share components or mathematical terms; the strongest correlations included WHtR with BRI (rho = 1.00), TyG with AIP (rho = 0.95), and BMI with TyG-BMI (rho = 0.94). Exploratory all-indices VIF diagnostics showed marked collinearity for mathematically related indices, confirming that these indices should not be interpreted as mutually independent predictors in a single joint model. No independent ultrasound-defined fatty liver variable was present in the uploaded analytic dataset; therefore, fatty liver was not analyzed as a study outcome.

### 3.3 Adjusted associations with adverse CKM phenotype

**Figure 5:**
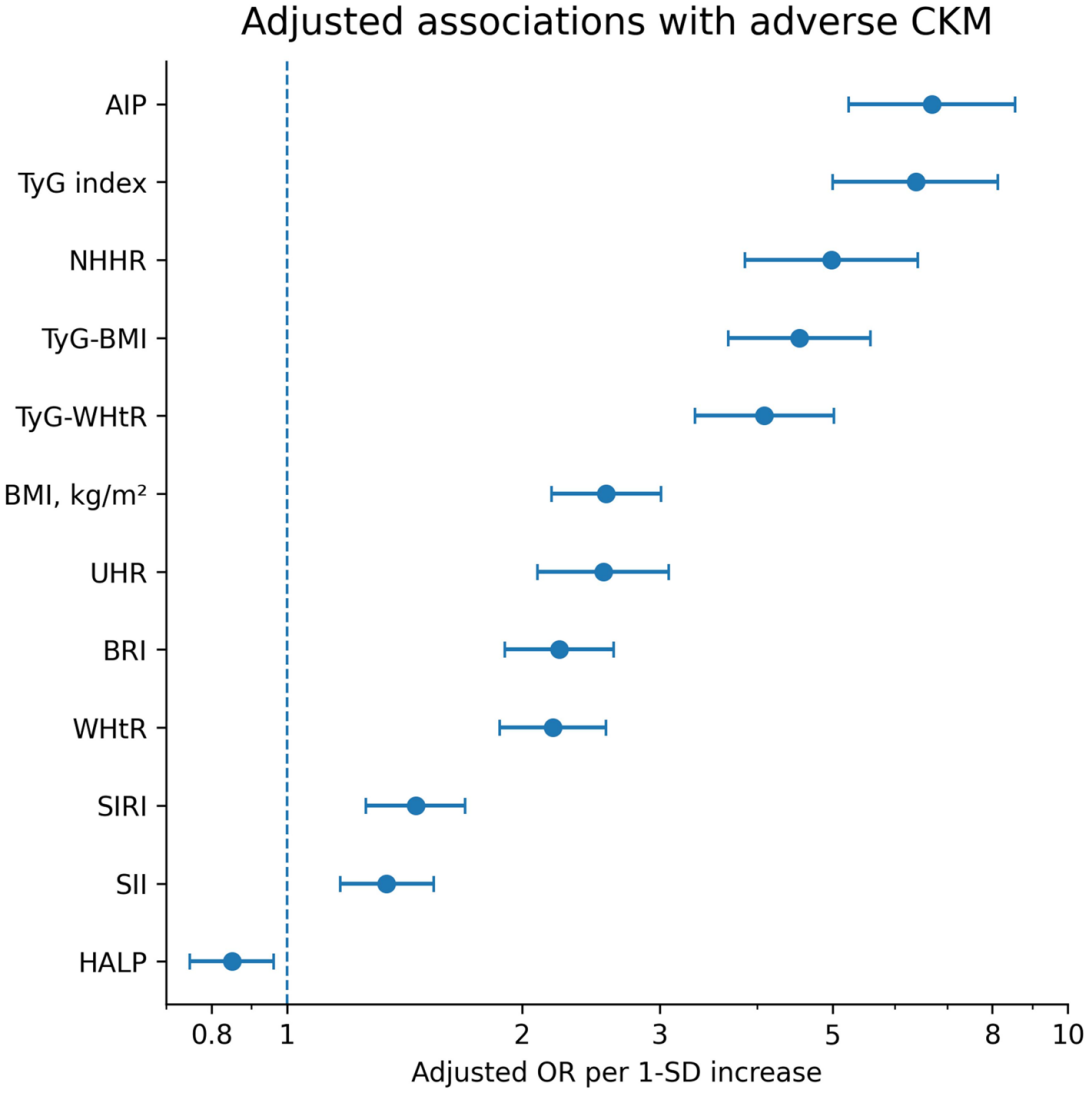
Forest plot of lifestyle-adjusted odds ratios per 1-*SD* increment in each index.

In logistic regression analyses adjusted for age, sex, smoking, alcohol use, exercise frequency, and sleep duration, several indices showed strong associations with adverse CKM phenotype. The strongest associations were observed for AIP (OR per 1-SD, 6.70; 95% CI, 5.24–8.56; *p* < 0.01), TyG index (OR, 6.38; 95% CI, 5.00–8.13; *p* < 0.01), NHHR (OR, 4.97; 95% CI, 3.86–6.41; *p* < 0.01), TyG-BMI (OR, 4.53; 95% CI, 3.67–5.58; *p* < 0.01), and TyG-WHtR (OR, 4.08; 95% CI, 3.33–5.01; *p* < 0.01). HALP showed an inverse association (OR, 0.85; 95% CI, 0.75–0.96; *p* < 0.01). Winsorization sensitivity analyses at the 1st and 99th percentiles showed materially similar estimates for the leading indices (AIP OR, 6.56; TyG OR, 6.29 per 1-SD increment), suggesting that the large ORs were not driven solely by extreme values. Regression results are shown in Table 3 and Figure 5.

**Table 3:** Adjusted associations of composite indices with adverse CKM phenotype.

Index	Model 1			Model 2		
	OR (95% CI)	*P*-value	AIC	OR (95% CI)	*P*-value	AIC
BMI, kg/m^2^	2.57 (2.19–3.01)	<0.01	1302.28	2.56 (2.18–3.01)	<0.01	1315.23
WHtR	2.20 (1.88–2.57)	<0.01	1352.68	2.19 (1.87–2.56)	<0.01	1364.89
TyG index	6.27 (4.94–7.97)	<0.01	1105.83	6.38 (5.00–8.13)	<0.01	1119.71
TyG-BMI	4.52 (3.67–5.56)	<0.01	1170.63	4.53 (3.67–5.58)	<0.01	1185.38
TyG-WHtR	4.09 (3.34–5.01)	<0.01	1206.25	4.08 (3.33–5.01)	<0.01	1221.07
BRI	2.24 (1.91–2.63)	<0.01	1351.66	2.23 (1.90–2.62)	<0.01	1363.97
AIP	6.58 (5.16–8.37)	<0.01	1087.59	6.70 (5.24–8.56)	<0.01	1101.35
NHHR	4.86 (3.79–6.25)	<0.01	1249.47	4.98 (3.86–6.42)	<0.01	1260.07
UHR	2.48 (2.05–3.00)	<0.01	1360.62	2.54 (2.09–3.08)	<0.01	1370.15
SII	1.37 (1.19–1.57)	<0.01	1444.92	1.34 (1.17–1.54)	<0.01	1456.27
SIRI	1.47 (1.27–1.70)	<0.01	1435.76	1.46 (1.26–1.69)	<0.01	1447.33
HALP	0.84 (0.74–0.96)	<0.01	1458.52	0.85 (0.75–0.96)	<0.01	1467.91

Odds ratios are reported per 1-SD increase in each index. Model 1 adjusted for age and sex. Model 2 adjusted for age, sex, smoking status, alcohol status, exercise frequency, and sleep duration.

### 3.4 Discrimination of individual indices

**Figure 6:**
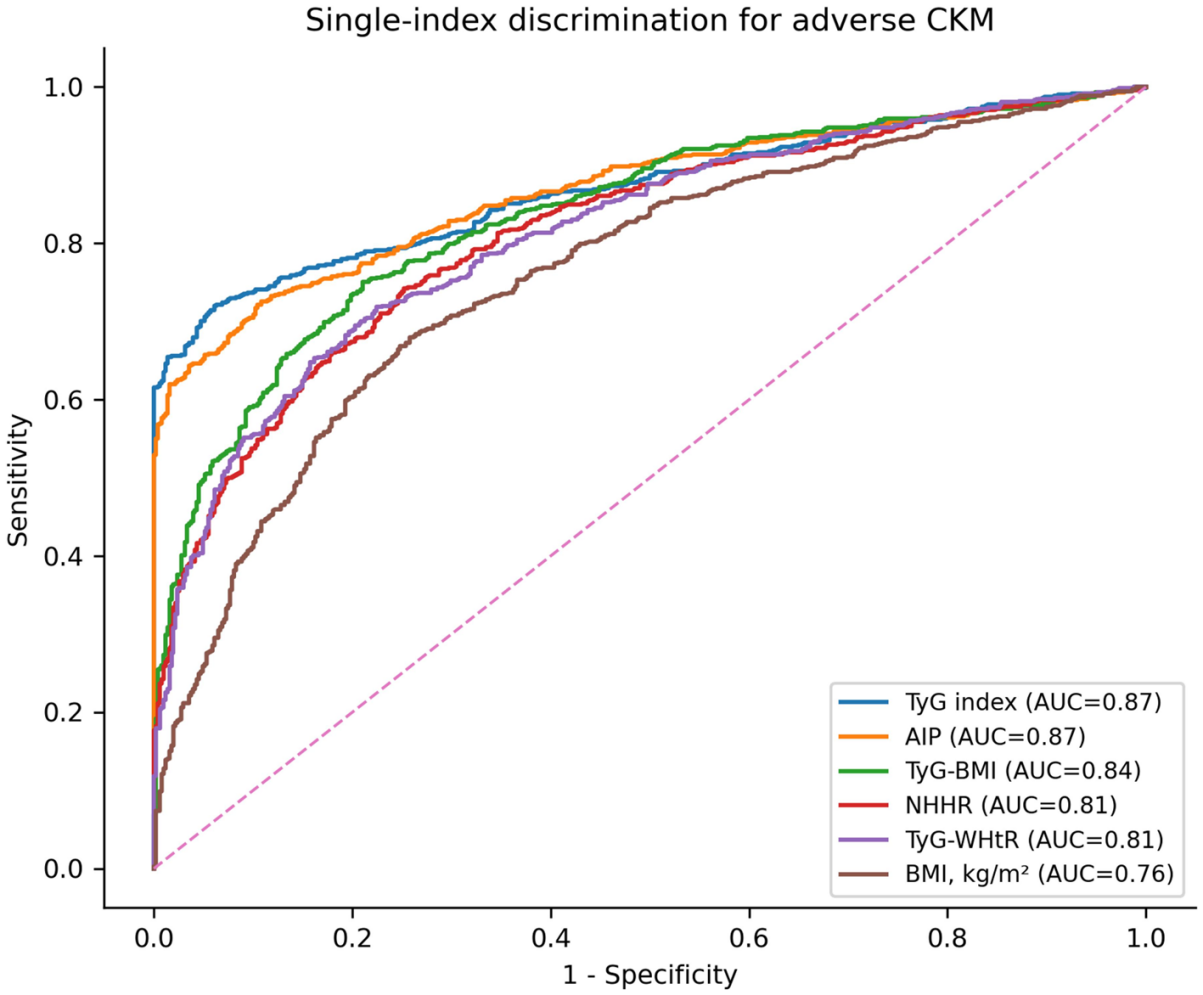
ROC curves for the highest-performing individual indices. AUC confidence intervals are provided in Table 4, and selected DeLong comparisons are provided in Supplementary Table 3.

Single-index ROC analyses showed that TyG and AIP had similarly high discrimination for adverse CKM phenotype, with AUCs of 0.87 (95% CI, 0.85–0.89; *p* < 0.01) for both. DeLong testing showed no statistically significant difference between TyG and AIP (AUC difference, 0.002; *p* = 0.625). TyG had higher discrimination than TyG-BMI (AUC difference, 0.035; *p* < 0.001), and AIP had higher discrimination than NHHR (AUC difference, 0.055; *p* < 0.001). TyG-BMI had an AUC of 0.84 (95% CI, 0.81–0.86; *p* < 0.01), followed by NHHR and TyG-WHtR (both AUC, 0.81; p < 0.01). BMI showed moderate discrimination (AUC, 0.76; 95% CI, 0.74–0.79; *p* < 0.01), whereas inflammatory indices showed lower discrimination. HALP had limited discrimination (AUC, 0.53; 95% CI, 0.49–0.56; *p* = 0.10). The optimal cutoffs in Table 4 were internally derived by the Youden index and should be interpreted as exploratory rather than clinically actionable thresholds. Full ROC results are presented in Table 4 and Figure 6; selected DeLong comparisons are shown in Supplementary Table 3.

**Table 4:** Discrimination of individual indices for adverse CKM phenotype.

Index	Direction associated with adverse CKM	AUC (95% CI)	*P*-value	Optimal cutoff	Sensitivity	Specificity
TyG index	Higher	0.87 (0.85–0.89)	<0.01	8.80	0.72	0.94
AIP	Higher	0.87 (0.85–0.89)	<0.01	0.07	0.73	0.89
TyG-BMI	Higher	0.84 (0.81–0.86)	<0.01	207.6	0.75	0.79
NHHR	Higher	0.81 (0.79–0.84)	<0.01	2.75	0.74	0.75
TyG-WHtR	Higher	0.81 (0.79–0.84)	<0.01	4.40	0.72	0.78
BMI, kg/m^2^	Higher	0.76 (0.74–0.79)	<0.01	24.09	0.68	0.74
UHR	Higher	0.73 (0.70–0.75)	<0.01	264.44	0.63	0.71
WHtR	Higher	0.71 (0.68–0.74)	<0.01	0.51	0.67	0.68
BRI	Higher	0.71 (0.68–0.74)	<0.01	3.53	0.67	0.68
SIRI	Higher	0.63 (0.59–0.66)	<0.01	0.75	0.58	0.61
SII	Higher	0.61 (0.58–0.64)	<0.01	471.79	0.51	0.66
HALP	Lower	0.53 (0.49–0.56)	0.1	62.23	0.80	0.27

AUC confidence intervals were calculated using large-sample variance approximation. Optimal cutoffs were identified by the Youden index and are internally derived exploratory cutoffs rather than clinically actionable thresholds. For HALP, lower values were associated with adverse CKM.

### 3.5 Non-linear associations and machine-learning models

Restricted cubic spline analyses supported statistically significant overall associations for selected lipid-insulin and lipid-metabolic indices, including AIP, TyG, NHHR, and UHR (all *p* values reported in Table 1 and Figure 2). Consistent with the Methods and the Figure 2 caption, the RCS curves were centered at the median and displayed with 95% CIs over the data-dense central range of each index to keep the curves and confidence bands within a reasonable visual scale. Embedded histograms and rug plots show where observations were concentrated. Within these displayed data-dense ranges, the odds of adverse CKM increased progressively as AIP, TyG, NHHR, and UHR increased; however, the sparse upper tails were not used to define discrete non-linear thresholds.

**Figure 7:**
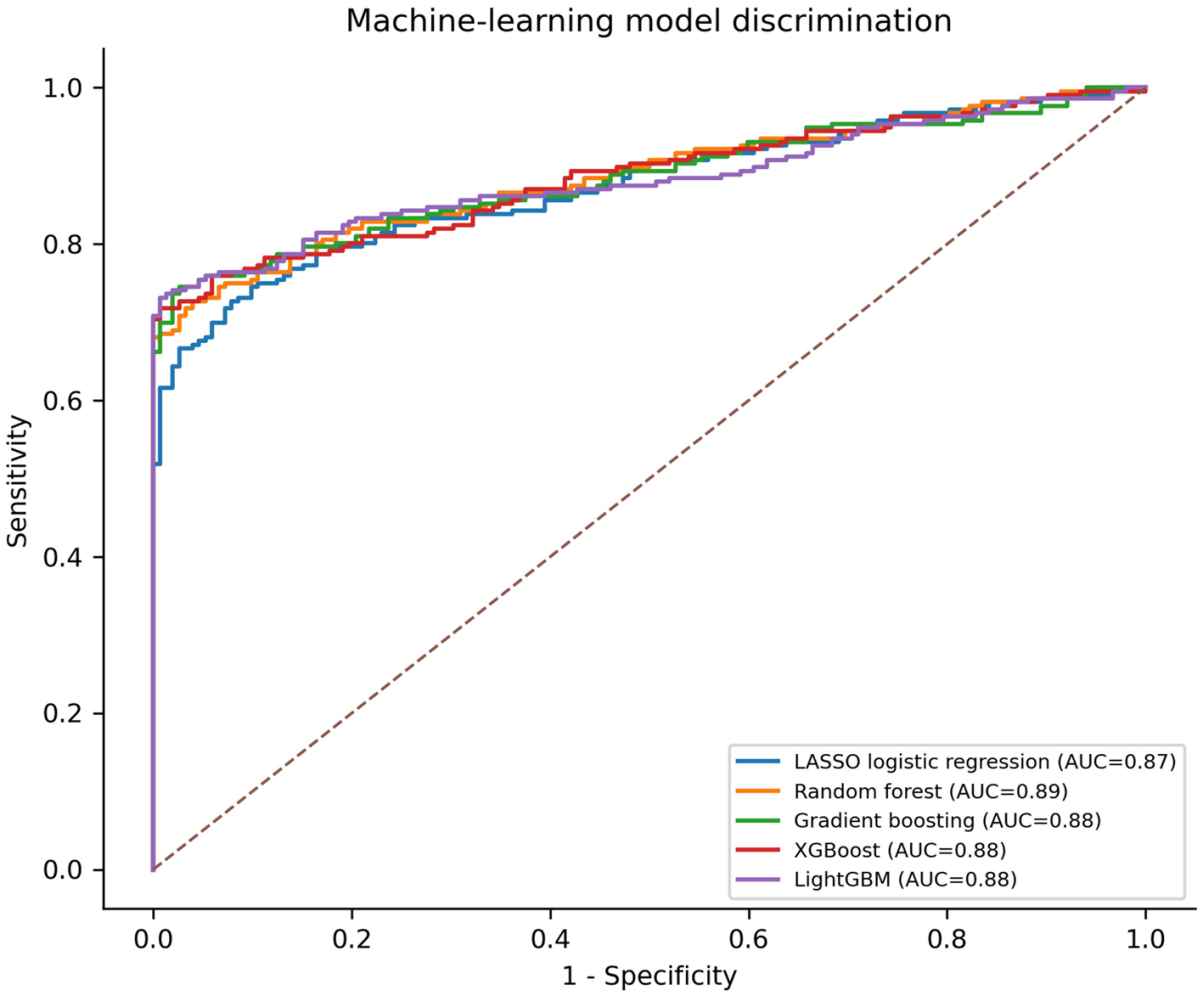
ROC curves for machine-learning models in the held-out testing set.

**Figure 8:**
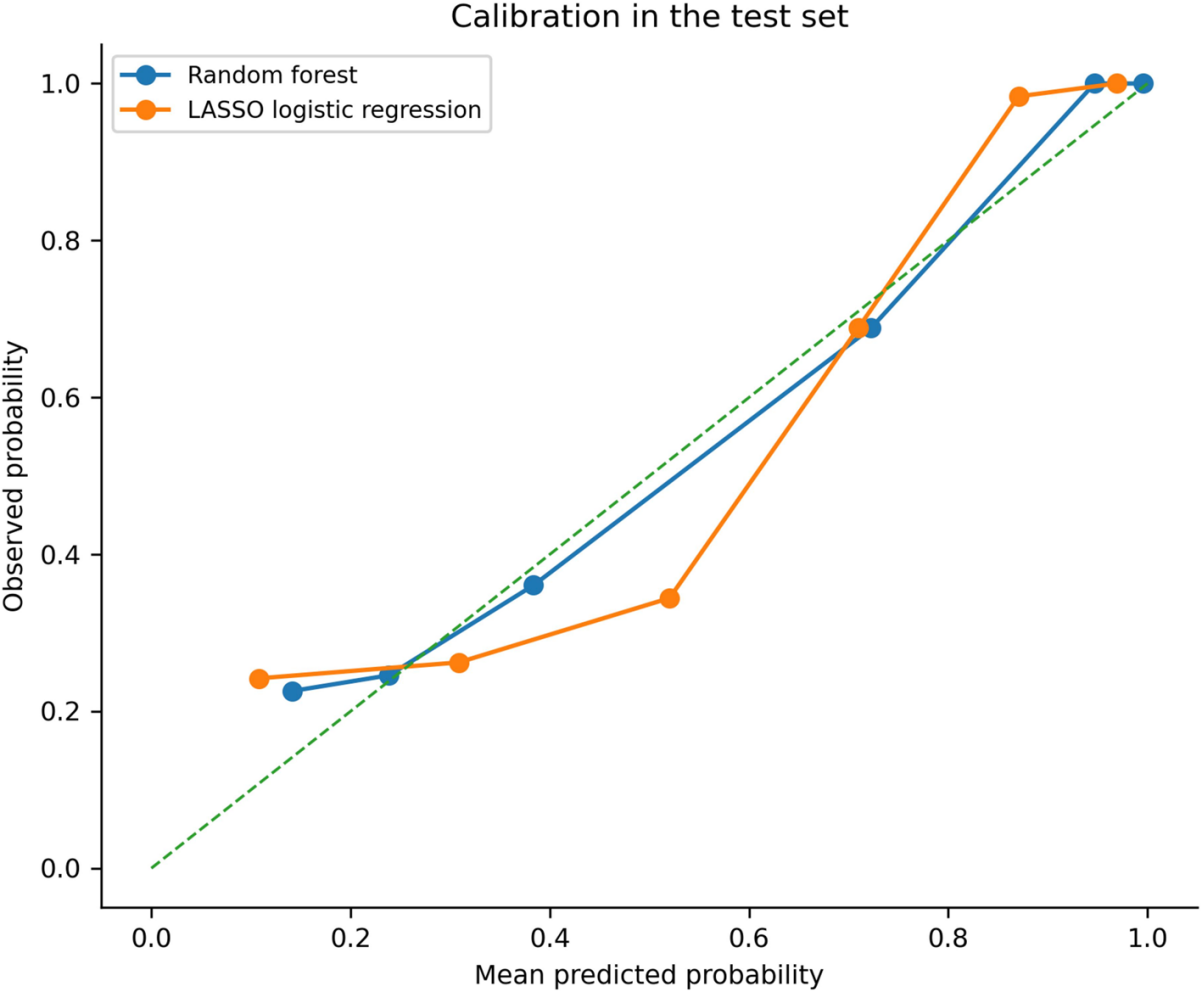
Calibration curves for the best-performing model and comparator model.

**Figure 9:**
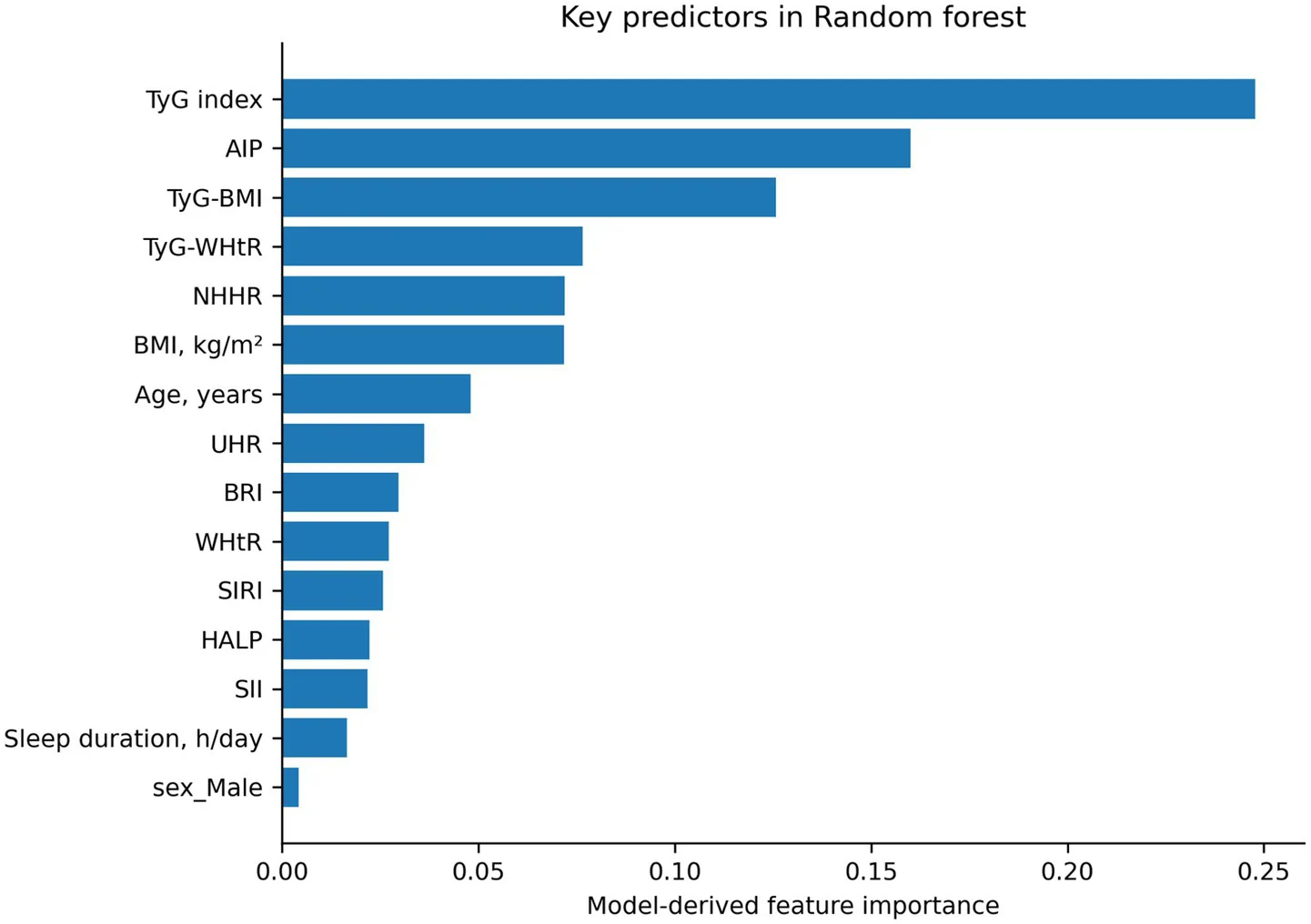
Model-derived feature importance for the best-performing algorithm.

In machine-learning analyses using a stratified 70:30 train-test split, random forest achieved the best test-set performance with AUC 0.89 (0.85–0.92) (*p* < 0.01), PR-AUC 0.94, accuracy 0.82, sensitivity 0.79, specificity 0.86, and Brier score 0.13. Model-derived feature-importance analysis identified lipid-insulin resistance and lipid-ratio indices as dominant predictors. However, the incremental discrimination over the strongest single indices was modest (random forest AUC, 0.89 vs. AIP and TyG AUCs, 0.87), and the machine-learning findings should be viewed as exploratory internal performance rather than evidence of deployable clinical superiority. Model performance is summarized in Table 5, with ROC, calibration, and importance plots shown in Figures 7–9.

**Table 5:** Machine-learning model performance in the held-out testing set.

Model	AUC (95% CI)	*P*-value	PR-AUC	Accuracy	Sensitivity	Specificity	Precision	F1-score	Brier score
Random forest	0.89 (0.85–0.92)	<0.01	0.94	0.82	0.79	0.86	0.89	0.84	0.13
XGBoost	0.88 (0.85–0.92)	<0.01	0.94	0.82	0.78	0.88	0.9	0.84	0.12
Gradient boosting	0.88 (0.85–0.92)	<0.01	0.94	0.82	0.79	0.88	0.9	0.84	0.12
LightGBM	0.88 (0.85–0.92)	<0.01	0.94	0.82	0.78	0.87	0.89	0.83	0.13
LASSO logistic regression	0.87 (0.84–0.91)	<0.01	0.93	0.79	0.82	0.74	0.82	0.82	0.14

Models used age, sex, lifestyle variables, and composite indices. Direct CKM component diagnoses and outcome labels were excluded from features. *P*-values correspond to AUC greater than 0.50. Performance estimates are based on a single internal stratified train-test split and should be interpreted as exploratory until externally validated.

Sex-stratified sensitivity analyses showed directionally consistent discrimination in both women and men. In women, TyG and AIP had AUCs of 0.82 (95% CI, 0.78–0.85) and 0.81 (95% CI, 0.77–0.84), respectively. In men, TyG and AIP had AUCs of 0.91 (95% CI, 0.88–0.93) and 0.91 (95% CI, 0.89–0.93), respectively. These analyses were considered exploratory and are summarized in Supplementary Table 4.

## 4 Discussion

In this single-center hospital-based cross-sectional study of 1,226 adults undergoing routine health examinations, adverse CKM phenotype was common, affecting 58.56% of participants. However, this adverse phenotype was overwhelmingly stage 2: 693 of 718 adverse CKM cases (96.52%) were stage 2, whereas stages 3 and 4 were uncommon. Thus, our findings primarily identify a prevalent stage 2-dominant CKM-related metabolic-risk phenotype rather than advanced CKM disease or future cardiovascular-kidney outcomes. Using only variables obtainable from routine physical examination, standard fasting biochemistry, complete blood count, and a brief questionnaire, we found that lipid-insulin resistance and lipid-metabolic composite indices were strongly associated with adverse CKM phenotype. AIP and TyG were similarly high-performing individual indices, each achieving an AUC of approximately 0.87, while TyG-BMI, TyG-WHtR, and NHHR also showed robust associations and discrimination.

These findings are consistent with the biological architecture of CKM syndrome. The AHA framework emphasizes that metabolic dysfunction, kidney impairment, and cardiovascular risk do not develop in isolation but reinforce one another through insulin resistance, ectopic adiposity, atherogenic dyslipidemia, inflammation, endothelial dysfunction, and neurohormonal activation (1, 2). In this context, a composite index may outperform a single raw biomarker because it captures a pathophysiological axis rather than an isolated laboratory abnormality.

The TyG index performed particularly well, which is biologically plausible because it combines fasting triglycerides and glucose-two routine markers that jointly approximate insulin resistance. Insulin resistance is a central driver of hypertriglyceridemia, impaired glucose regulation, hypertension, visceral fat accumulation, and kidney-metabolic stress. Nevertheless, this strong performance must be interpreted cautiously because triglycerides and glucose are closely related to stage 2 CKM criteria; similarly, AIP includes triglycerides and HDL-C, which overlap with dyslipidemic components of the CKM framework. This predictor-outcome overlap means that AIP and TyG should be interpreted as compact summary markers of prevalent CKM-related metabolic burden, not as independent etiologic predictors or validated prognostic tools. Our results therefore extend prior validation studies of TyG as an insulin-resistance surrogate only in the context of identifying prevalent CKM stage > = 2 phenotype in a real-world health examination setting (4, 5). The performance of TyG-WHtR is also consistent with recent cohort evidence linking combined glycemic-lipid and central-adiposity indices with cardiovascular outcomes and mortality (30, 31), but our study itself did not assess longitudinal events.

AIP showed one of the strongest adjusted associations and performed similarly to TyG in discrimination. DeLong testing did not show a statistically meaningful difference between AIP and TyG, and we therefore describe them as similarly high-performing rather than ranking one above the other. This finding highlights the importance of atherogenic dyslipidemia in CKM staging. AIP integrates triglyceride elevation and HDL-C reduction, two abnormalities closely linked to small dense LDL particles, remnant cholesterol burden, insulin resistance, and vascular risk (11, 12, 32). In this dataset, AIP may summarize lipid quality and particle-related risk, but its diagnostic performance should still be interpreted in light of overlap between its components and CKM stage-defining metabolic abnormalities.

NHHR and UHR provided additional information on lipid-metabolic and uric-acid-related risk. NHHR reflects the balance between atherogenic non-HDL cholesterol and protective HDL-C and may be more comprehensive than LDL-C alone for routine screening (13, 14). UHR links hyperuricemia with low HDL-C, potentially capturing oxidative stress, purine metabolism, and metabolic inflammation (15, 16). Although UHR discrimination was lower than TyG and AIP, its association remained statistically robust and clinically relevant because uric acid is routinely measured in many Chinese health examination programs.

Inflammation-related indices, including SII and SIRI, were associated with adverse CKM despite their individual components not being part of stage 2 CKM definitions (17–19). This makes them complementary signals rather than mere reflections of diagnostic lipid or glycemic criteria. However, their AUCs were substantially lower than those of lipid-insulin indices, suggesting that low-grade inflammation may accompany adverse CKM phenotypes but may not serve as a strong standalone screening tool in a relatively general health-check population. HALP was inversely associated with adverse CKM in adjusted regression, but its single-index AUC was limited, implying that nutrition-inflammation balance may be more useful as a complementary feature than as an independent screening tool (20, 21).

The machine-learning analysis suggests that routine variables can classify prevalent adverse CKM phenotype with good internal performance. Models combining demographics, questionnaire variables, and composite biomarkers achieved strong discrimination in held-out testing, and feature-importance analysis consistently prioritized lipid-insulin resistance and lipid-ratio indices. Importantly, direct CKM component diagnoses were excluded from the model feature set to reduce circularity. However, the clinical incremental value of machine learning appears modest because random forest improved AUC only from approximately 0.87 for AIP/TyG alone to 0.89. From an implementation perspective, a simple index may be preferable when the goal is rapid preliminary risk summarization, whereas machine learning may be more useful for integrated EHR-based ranking after external validation. The interpretability-focused design is aligned with current reporting and implementation principles for clinical prediction models and biomedical machine learning (22–27), but these internally evaluated models are not ready for deployment.

Several limitations should be considered. First, the cross-sectional design precludes causal inference and cannot determine whether these indices predict incident diabetes, CKD progression, cardiovascular events, heart failure, mortality, or CKM stage progression. Second, the cohort was derived from a single hospital health examination center and used complete-case inclusion based on available data, so selection bias is possible and multicenter external validation is required. Third, CKM classification was based on available health examination records and physician diagnoses; albuminuria/UACR and independent imaging-defined fatty liver data were unavailable, which may have affected kidney-risk stratification and AHA CKM staging. Fourth, hyperuricemia was recorded as an additional cardiometabolic comorbidity but was not used as an independent AHA CKM stage-defining criterion. Fifth, because adverse CKM was dominated by stage 2 and stages 3–4 were rare, the results should not be generalized to advanced CKM populations. Sixth, AIP and TyG share components with metabolic criteria used in stage 2 CKM, creating predictor-outcome overlap that may inflate apparent discrimination. Seventh, multiple correlated indices were evaluated without formal multiplicity correction; therefore, the analyses should be regarded as exploratory and hypothesis-generating. Finally, machine-learning models were evaluated using a single internal train-test split without repeated cross-validation or external validation, and should be prospectively validated before clinical implementation.

## 5 Conclusion

Among adults undergoing routine health examinations, adverse CKM phenotype was common but predominantly represented stage 2 disease. AIP and TyG were similarly high-performing low-cost summary markers for prevalent CKM-related metabolic risk, while machine-learning models provided only modest incremental discrimination over these simple indices. These findings may support preliminary risk stratification and prioritization for further clinical evaluation in hospital-based health examination settings, but they require prospective multicenter and longitudinal validation before clinical implementation.

## Data Availability

The original contributions presented in the study are included in the article/Supplementary material, further inquiries can be directed to the corresponding author.
