# Supplementary material for: Routine metabolic and nutrition-related composite indices for identifying adverse cardiovascular-kidney-metabolic phenotypes: a cross-sectional study
Source: Front Nutr. 2026 Aug 21;13:1872579. doi: 10.3389/fnut.2026.1872579 (PMC13541932; doi:10.3389/fnut.2026.1872579)
Supplement: Supplementary file 1 [file Table_1.docx]

**Supplementary Tables**

**Supplementary Table 1. Operational CKM stage definitions used in this study**

| **CKM stage** | **Operational definition** | **Available-data considerations in this study** |
| --- | --- | --- |
| Stage 0 | No CKM risk factors: normal adiposity, blood pressure, glycemia, and lipid status; no CKD; no subclinical or clinical CVD. | Extracted from physician-assigned stage/diagnosis when no risk factors were recorded. |
| Stage 1 | Excess or dysfunctional adiposity or abnormal glucose metabolism without other major metabolic risk factors or CKD. Adiposity thresholds included BMI >=25 kg/m² (Asian threshold >=23 kg/m²), waist circumference >=88/102 cm in women/men (Asian threshold >=80/90 cm), fasting glucose 100-124 mg/dL, or HbA1c 5.7%-6.4%. | Stage 1 was based on physician diagnosis and available anthropometric/glycemic data. |
| Stage 2 | Metabolic risk factors or CKD, including hypertension, hypertriglyceridemia/dyslipidemia, metabolic syndrome, diabetes, or moderate-to-high-risk CKD. | Hyperuricemia was not used as an independent stage 2 criterion; it was recorded as an additional comorbidity. Albuminuria/UACR was unavailable. |
| Stage 3 | CKM with subclinical CVD or high-risk states, including subclinical ASCVD, subclinical heart failure, very-high-risk CKD (e.g., CKD G4-G5/KDIGO very high risk), or high predicted CVD risk. | Extracted from physician diagnosis/recorded risk status when available; UACR was unavailable. |
| Stage 4 | CKM with clinical CVD, including coronary heart disease, heart failure, stroke, peripheral artery disease, atrial fibrillation, or related clinical CVD. Stage 4A indicates no kidney failure, and stage 4B indicates kidney failure. | Extracted from physician-diagnosed clinical CVD and kidney-failure information when available. |

*Note: CKM staging followed the AHA conceptual framework but was operationalized according to complete data availability and physician diagnoses in the health examination records. Researchers extracted the diagnoses and did not impute unavailable AHA components.*

**Supplementary Table 2. Candidate indices, formulas, required variables, and intended biological interpretation**

| **Index** | **Formula and required variables/units** | **Intended biological interpretation** |
| --- | --- | --- |
| BMI | Weight (kg) / height² (m²) | General adiposity |
| WHtR | Waist circumference / height using identical length units | Central adiposity |
| TyG index | ln [fasting triglycerides (mg/dL) × fasting plasma glucose (mg/dL) / 2]; mmol/L values converted when required | Insulin resistance / glycemic-lipid stress |
| TyG-BMI | TyG index × BMI | Insulin resistance combined with general adiposity |
| TyG-WHtR | TyG index × WHtR | Insulin resistance combined with central adiposity |
| BRI | 364.2 - 365.5 × sqrt{1 - [(waist circumference/2π)² / (0.5 × height)²]}; waist and height in consistent units | Body shape / central adiposity geometry |
| AIP | log10(triglycerides / HDL-C), using consistent lipid units | Atherogenic dyslipidemia / lipoprotein particle-related risk |
| NHHR | (total cholesterol - HDL-C) / HDL-C | Atherogenic cholesterol burden relative to HDL-C |
| UHR | Serum uric acid / HDL-C | Purine metabolism, oxidative stress, and lipid-metabolic imbalance |
| SII | Platelet count × neutrophil count / lymphocyte count | Systemic immune-inflammation |
| SIRI | Neutrophil count × monocyte count / lymphocyte count | Systemic inflammatory response |
| HALP | Hemoglobin × albumin × lymphocyte count / platelet count | Nutrition-immune reserve and inflammation balance |

**Supplementary Table 3. DeLong comparisons among selected top-performing individual indices**

| **Comparison** | **AUCs compared** | **AUC difference** | **P value / interpretation** |
| --- | --- | --- | --- |
| TyG index vs AIP | 0.87 vs 0.87 | <0.01 | 0.63 |
| TyG index vs TyG-BMI | 0.87 vs 0.84 | 0.04 | <0.01 |
| AIP vs NHHR | 0.87 vs 0.81 | 0.06 | <0.01 |
| AIP vs TyG-BMI | 0.87 vs 0.84 | 0.03 | <0.01 |

**Supplementary Table 4. Sex-stratified discrimination of selected individual indices**

| **Index** | **Women AUC (95% CI)** | **Men AUC (95% CI)** |
| --- | --- | --- |
| TyG index | 0.82 (0.78-0.85) | 0.91 (0.88-0.93) |
| AIP | 0.81 (0.77-0.84) | 0.91 (0.89-0.93) |
| TyG-BMI | 0.80 (0.76-0.84) | 0.86 (0.83-0.89) |
| NHHR | 0.77 (0.73-0.80) | 0.84 (0.81-0.87) |
| TyG-WHtR | 0.80 (0.76-0.83) | 0.84 (0.81-0.87) |

*Note: Sex-stratified analyses were exploratory sensitivity analyses and were not intended to establish sex-specific clinical thresholds.*
